# New insights into BaP-induced toxicity: role of major metabolites in transcriptomics and contribution to hepatocarcinogenesis

**DOI:** 10.1007/s00204-015-1572-z

**Published:** 2015-08-04

**Authors:** Terezinha Souza, Danyel Jennen, Joost van Delft, Marcel van Herwijnen, Soterios Kyrtoupolos, Jos Kleinjans

**Affiliations:** Department of Toxicogenomics, Maastricht University, 6229 ER Maastricht, The Netherlands; Institute of Biology, Medicinal Chemistry and Biotechnology, National Hellenic Research Foundation, 11635 Athens, Greece; Department of Toxicogenomics, Maastricht University, Universiteitsingel 50, 6200 MD Maastricht, The Netherlands

**Keywords:** Hepatocellular carcinoma, Transcriptomics, Benzo(a)pyrene, Hepatocarcinogenesis, BaP metabolites

## Abstract

Benzo(a)pyrene (BaP) is a ubiquitous carcinogen resulting from incomplete combustion of organic compounds and also present at high levels in cigarette smoke. A wide range of biological effects has been attributed to BaP and its genotoxic metabolite BPDE, but the contribution to BaP toxicity of intermediary metabolites generated along the detoxification path remains unknown. Here, we report for the first time how 3-OH–BaP, 9,10-diol and BPDE, three major BaP metabolites, temporally relate to BaP-induced transcriptomic alterations in HepG2 cells. Since BaP is also known to induce AhR activation, we additionally evaluated TCDD to source the expression of non-genotoxic AhR-mediated patterns. 9,10-Diol was shown to activate several transcription factor networks related to BaP metabolism (AhR), oxidative stress (Nrf2) and cell proliferation (HIF-1α, AP-1) in particular at early time points, while BPDE influenced expression of genes involved in cell energetics, DNA repair and apoptotic pathways. Also, in order to grasp the role of BaP and its metabolites in chemical hepatocarcinogenesis, we compared expression patterns from BaP(-metabolites) and TCDD to a signature set of approximately nine thousand gene expressions derived from hepatocellular carcinoma (HCC) patients. While transcriptome modulation by TCDD appeared not significantly related to HCC, BaP and BPDE were shown to deregulate metastatic markers via non-genotoxic and genotoxic mechanisms and activate inflammatory pathways (NF-κβ signaling, cytokine–cytokine receptor interaction). BaP also showed strong repression of genes involved in cholesterol and fatty acid biosynthesis. Altogether, this study provides new insights into BaP-induced toxicity and sheds new light onto its mechanism of action as a hepatocarcinogen.

**Electronic supplementary material:**

The online version of this article (doi:10.1007/s00204-015-1572-z) contains supplementary material, which is available to authorized users.

## Introduction

Polycyclic aromatic hydrocarbons (PAHs) represent a large group of environmental pollutants formed after incomplete combustion of organic material, and many of them suspected or unequivocally acknowledged as human carcinogens (IARC 1983). Among PAHs, benzo(a)pyrene (BaP) stands out as a prototypical human carcinogen, found at high levels in cigarette smoke (CS), in polluted water, soil and air and also in high-temperature processed meats (IARC 2012).

BaP contributes to approximately 50 % of the total carcinogenic potential of the PAH group: occupational exposure for instance is associated with lung, bladder, oral cavity, esophagus, hematolymphatic, skin, lip, pharynx and larynx cancers (IARC 2012). Such risks for human health motivated the discovery of its main toxic mechanisms, which relate to transcriptional activation of the aryl hydrocarbon receptor (AhR) and many other transcription factor families, as well as to the induction of oxidative stress, mitogenic signaling and DNA adduction by its ultimate carcinogenic metabolite benzo(a)pyrene-7,8-dihydrodiol 9,10-epoxide BPDE (Miller and Ramos 2001; van Delft et al. 2010). Molecular aspects and gene transcription modulation were largely unraveled due to high-throughput techniques applied to several cell models, including lung, breast, skin and especially liver (Wilkening et al. 2003; Hockley et al. 2006; van Delft et al. 2010; Jetten et al. 2013).

However, these studies are mainly focused on the overall response to BaP or at most BPDE, while intermediary metabolites are disregarded with respect to their potential contribution to BaP toxicity. Therefore, the main goal of our study is to perform time-dependent analysis of the induction by BaP in comparison with its major metabolites of whole-genome gene expression. Thus, we selected the non-genotoxic (NGTX) metabolites 3-hydroxybenzo(a)pyrene (3-OH–BP) and benzo(a)pyrene-9,10-dihydrodiol (9,10-diol) and the genotoxic (GTX) metabolite epoxide benzo(a)pyrene-7,8-dihydrodiol 9,10-epoxide (BPDE). Also, in order to extract biological responses detached from DNA damage routes, we propose a comparative analysis by also evaluating the carcinogen 2,3,7,8-tetrachlorodibenzodioxin (TCDD), a non-genotoxicant but also a potent AhR agonist. We hypothesized that a portrayal of their respective effects will provide valuable and novel mechanistic information on BaP-induced carcinogenicity.

Besides its implication in several cancers, BaP may also be involved in the development of hepatocellular carcinoma (HCC) (El-Serag et al. 2008; Altekruse et al. 2009). Epidemiological studies of smoking cohorts with low prevalence of alcohol intake and viral infection show high incidence of HCC (Kuper et al. 2000; Farazi and DePinho 2006; Lee et al. 2009) and experimental data from BaP-exposed rodents show a causal relationship with the onset of liver tumors (IARC 2012). Thus, in order to investigate the molecular significance of (non-)genotoxic mechanisms of BaP exposure for hepatocarcinogenesis, we compared gene expression patterns from BaP(-metabolites) to a recently established in vivo signature set from HCC patients comprising approximately nine thousand genes (Caiment et al. 2014). For this, we exploited HepG2 cells, an easy-to-handle hepatoblastoma-derived cell line which has been shown to be capable of inducing phase I- and phase II-detoxification enzymes necessary for complete BaP metabolism and DNA adduct formation (Wilkening et al. 2003). Also, HepG2 has been described as a sensitive model for identifying and quantifying DNA-damaging properties of environmental and dietary agents (Knasmüller et al. 1998) and is able to efficiently discriminate GTX from NGTX compounds by gene expression profiling (van Delft et al. 2004; Magkoufopoulou et al. 2011).

## Materials and methods

### Cell culture and treatment

HepG2 cells purchased from American Type Culture Collection (ATCC, HB-8065) were cultured in minimum essential medium (MEM) supplemented with 1 % nonessential aminoacid, 1 % sodium pyruvate, 2 % penicillin/streptomycin and 10 % fetal bovine serum (FBS) in T25 culture flasks at 37 °C and 5 % CO_2_. After reaching confluence following 13–15 passages, cells were harvested and transferred into new culture flasks. The next day, the medium was replaced with fresh medium containing 3 µM BaP (CAS no. 50-32-8), 0.3 µM BPDE (CAS no. 55097-80-8), 3 µM 3-OH–BaP (CAS no. 13345-21-6), 3 µM 9,10-diol (CAS no. 62600-11-7) or 10 nM TCDD (CAS no. 1746-01-6) or vehicle control (DMSO, 0.5 %). Doses were selected based on IC_20_ values following MTT testing. The cells were exposed for 6, 12 and 18 h; thereafter, the medium was removed from the culture flasks, the media discarded and Trizol (Gibco/BRL) added for RNA and DNA isolation. Two independent experiments were conducted.

### Transcriptomic sample preparation and data analyses

#### RNA and DNA isolation

RNA was isolated from the Trizol solutions according to the manufacturer’s guidelines and purified using the RNeasy mini kit (Qiagen); the remaining phase was used for DNA isolation. RNA and DNA amounts were measured using a spectrophotometer, and RNA quality was determined by means of a BioAnalyzer (Agilent). Only RNA samples presenting distinct 18S and 28S peaks and RIN values higher than 8 were used for labeling and hybridization.

#### Labeling and hybridization

Labeling and hybridization of RNA samples were performed according to Agilent’s manual for microarrays (Agilent Technologies). Samples from BaP, BPDE, 3-OH–BaP, 9,10-diol or vehicle-treated cells were labeled by means of cyanine 3 (Cy3) or cyanine 5 (Cy5). Complementary RNA of the time-matched treated and control samples was applied on the Agilent 4 × 44 K human whole-genome microarray platform, hybridized and washed according to Agilent’s manual. Slides were scanned using a ScanArrayExpress (Packard Biochip Technologies). To correct for technical error and dye-related effects, for each biological replicate two hybridizations per time point with swapped Cy3 and Cy5 dyes were performed, resulting in 48 hybridizations. Further analyses were performed as single channel, i.e., data from each dye were analyzed separately to remove dye bias.

#### Image analysis and processing

The images obtained (16-bit tiff) were processed with GenePixPro software (Axon Instruments) to quantify spot intensities. Quality control and normalization were performed on these data using Bioconductor packages for R as follows: local background correction, flagging of bad spots, controls and spots with too low intensity, log transformation to base 2, quantile normalization and scaling to correct for differences between the used dyes. Genes with >30 % flags for either dye were excluded from further analysis, and missing values were imputed with K-nearest neighbor imputation (KNN, *n* = 15). Furthermore, for repeated genes on the microarray expression, values were merged by taking the median of these values.

Limma (linear model for microarray data) was used to generate lists of differentially expressed genes between control and treated samples, based on moderated t-statistics with FDR <0.05 and absolute fold change of 1.5. From each list, only genes with consistent direction of expression regulation among all replicates were selected. A union list containing genes significantly under- and over-expressed from all treatments was used for further analyses.

#### Occurrence of differentially expressed genes among treatments

In order to assess similarities among treatments and time points, we used VennMapper (Smid et al. 2003) to calculate *Z* scores based on the number of overlapping genes which have the same direction of regulation. From each sample, two separate files containing *Z* scores for up- and down-regulated genes were generated, allowing the selection of compounds with the most similar expression for each subset (i.e., *Z* score > 1.96).

#### Functional annotation and pathway analysis

From the selected genes, matching Entrez Gene IDs were used in an overrepresentation pathway analysis using ConsensusPathDB (Kamburov et al. 2013), a meta-database which integrates content from several pathway-finding sources. We selected pathways and GO terms with corrected Bonferroni *p* value lower than 0.05 and at least four candidate genes. Separate analyses for up- and down-regulated genes from each treatment were conducted.

### DNA adduct analysis

DNA adduct levels of BaP(-metabolite)-treated and control samples were quantified by ^32^P-postlabelling as described elsewhere (Godschalk et al. 1998). Results were expressed as DNA adducts/10^7^ nucleotides and presented as mean ± standard deviation of two experiments.

### Correlation analysis

Log 2-transformed DNA adduct levels were used for correlation analysis with gene expression values from 1913 genes present in the union list. Pearson correlation coefficients were calculated using GraphPad Prism v. 5.0; afterward, genes with coefficients higher than 0.5 and *p* value <0.01 were selected for pathway analysis as previously described.

## Results

### DNA adduct analysis

**Fig. 1:**
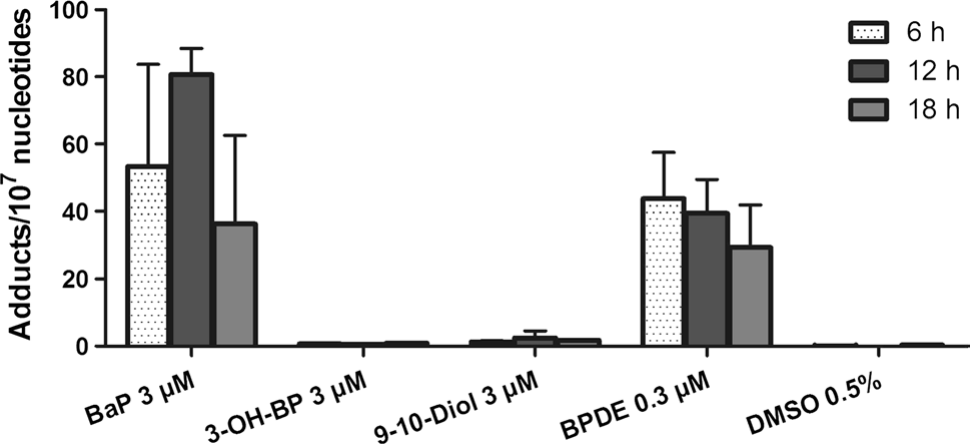
DNA adduct formation in HepG2 cells after exposure to BaP and its metabolites, using IC_20_ doses. Values are mean and standard deviation from two independent experiments

Adduct formation was observed only in BaP- and BPDE-exposed HepG2 cells (Fig. 1). Adduct levels reached their maximum after 12 h of BaP treatment and declined after 18 h. For BPDE, however, maximum levels were observed at the earliest time point (6 h).

### Alterations in gene expression in HepG2 following exposure to BaP and its metabolites

BaP and BPDE had the largest effect on the modulation of gene expression in HepG2 cells, as reflected by the number of differentially expressed genes (DEGs) at each exposure time point (Supplementary Data 1). While BPDE exerted the highest deregulation at the earliest time point (6 h; 519 genes), which declined gradually until a minimum was reached after 18 h (58 genes), BaP showed an inverse response, with an increasing DEGs number over exposure time reaching a maximum of 908 genes at time point 18 h. 9,10-Diol presented a temporal expression pattern similar to BaP, although to a lesser extent, regarding the number of DEGs. 3-OH-BP, however, did not alter expression of any gene after 6 h and <10 genes were modulated after 12 and 18 h.

**Fig. 2:**
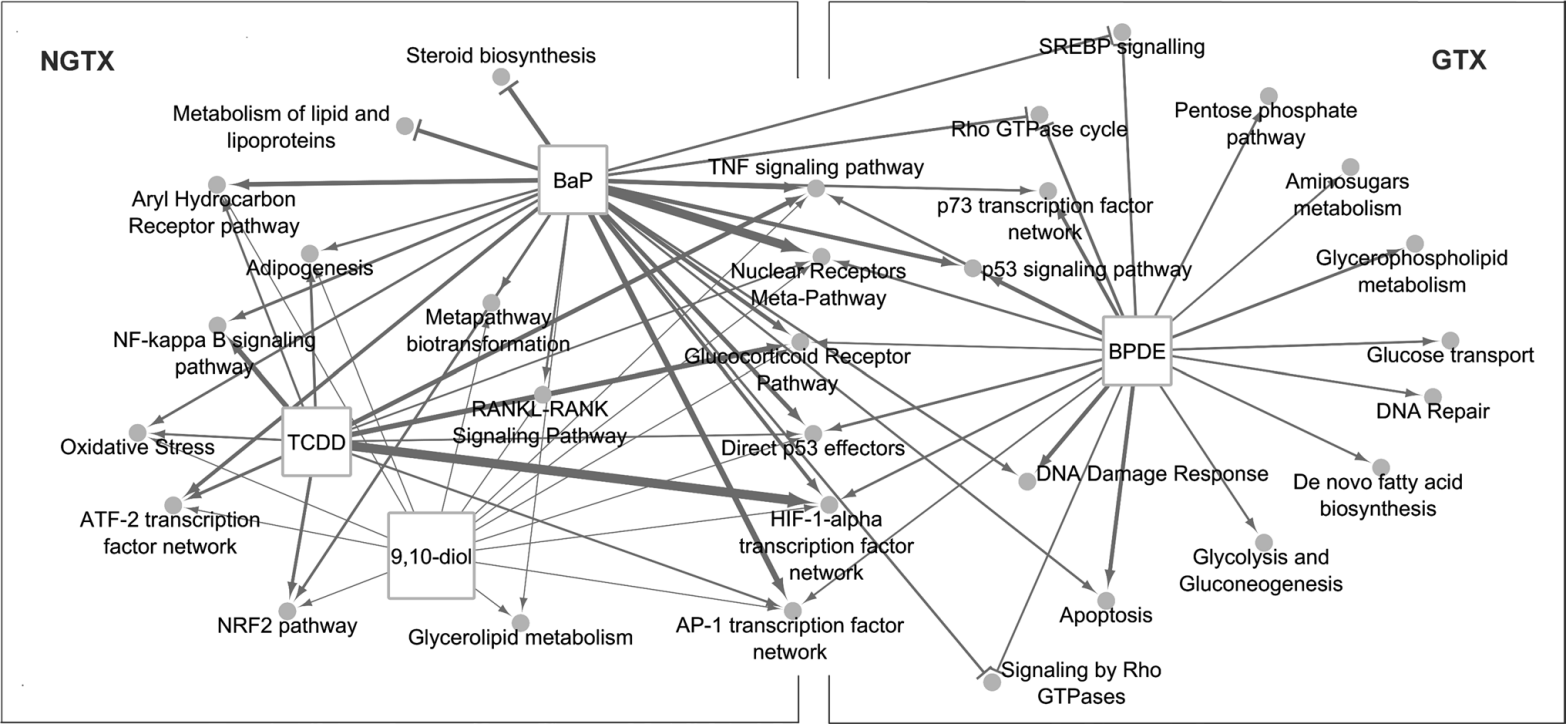
Main pathways affected by exposure to BaP and its NGTX metabolite, 9,10-dihydrodiol and its GTX metabolite BPDE, at least one time point tested (6, 12 or 18 h). Line thickness between nodes is relative to statistical significance. NGTX and GTX: pathways triggered by non-genotoxic and genotoxic metabolites, respectively

From each set of DEGs, up- and down-regulated genes were used to respectively retrieve over-represented pathways following BaP, 9,10-diol and BPDE treatments. An overview of deregulated pathways is represented in Fig. 2. A detailed description of deregulated pathways is available via the Supplementary Data 1.

Several TF networks were identified to be enriched at at least one exposure time point: AP-1, ATF-2, TNF-α, TGF-β, p53 and HIF-1α. Besides BaP, 9,10-diol was also capable of inducing the AhR transcriptional battery, as reflected by significant up-regulation of the AhR/ARNT targets *CYP1A1* and *NQO1*. Along with AhR, Nrf2 transcription factor was also activated by these compounds (enhancing *HMOX*-*1*, *GCLC* and *GCLM* expression). Oxidative stress signaling was activated by both BaP and 9,10-diol treatments, notably increasing *TXNRD1*, *SRXN1* and the aforementioned *HMOX*-*1*, among others. 9,10-Diol and BaP-altered gene expressions generally involved lipid metabolism (“adipogenesis,” “glycerolipid metabolism,” “metabolism of lipids and proteins”); the latter showed also a major effect on the repression of several genes specifically involved in cholesterol, steroid, triacylglycerides, phospho- and glycero-lipids metabolism.

At the earliest time point, BPDE exposure appeared to induce a unique set of pathways, including some involved in cell energetic metabolism, cell cycle and DNA repair—the latter only affected by BPDE treatment at this specific time point. At 12 and 18 h, BPDE exposure induced a set of pathways similar to those deregulated by BaP treatment. In both treatments, p53-regulated genes responsive to DNA damage stimulus such as *SESN1*, *CDKN1A* and *BTG2* were found to be up-regulated at at least one time point—while *CDKN1A* was increased at all three time points by both treatments. Expression of genes involved in nucleotide excision repair (e.g., *ERCC5*, *PCNA*) was only affected at the earliest time point of BPDE exposure. Apoptotic pathways were enriched by both treatments as well, as represented by the expression of pro-apoptotic genes (*FAS*, *TNFRSF10B*, *BAX*, *CASP8*) and the repression of the anti-apoptotic gene *BIRC5*. Anti-apoptotic response was also observed through the up-regulation of caspase inhibitor *BIRC3*.

Correlation analysis between DNA adducts and gene expression modulation resulted in 114 genes—52 positively and 62 negatively associated with DNA damage (Supplementary Data 1). Strong associations were observed for negative regulators of p53 signaling (*PPM1D*, *MDM2*), pro-apoptotic (*BBC3*) and antiproliferative (*BTG2*) genes. The strongest negative correlation was observed for *COMMD1*, a NF-κβ repressor.

### Gene expression equivalence between BaP(-metabolites) and TCDD

BaP exposure for 6 h significantly induced a large set of genes in common with either TCDD (after 12 h of exposure) and 9,10-diol (after 6 h of exposure), which belong to a gene cluster regulated in response to AhR and Nrf2 activation (see Supplementary Figure 1). TCDD and 9,10-diol deregulated processes linked to activation of the same TF batteries also at later time points, although to a lesser extent. The impact of DNA damage on gene expression (as observed by similarity scores with BPDE) is considerable in regard to gene expression, linked only to relatively late responses (12 and 18 h) and directed to a p53 effective response. Although the trend of *Z* scores distribution is very similar between up- and down-regulated genes among all compounds—including TCDD—in comparison with BaP, BPDE at the earliest time point again showed a very unique pattern. While upregulated genes were poorly related to BaP exposure, genes downregulated by BPDE showed a significant amount of overlap with 12 h BaP treatment, which was the largest cluster found among all comparisons. At 12 h and 18 h of exposure, although expression of AhR- and Nrf2-related features was still high and very similar to TCDD (mid and late time points), 9,10-diol showed a decreased induction of AhR genes and increased alterations of gene expressions associated with lipid metabolism and oxidative stress.

**Fig. 3:**
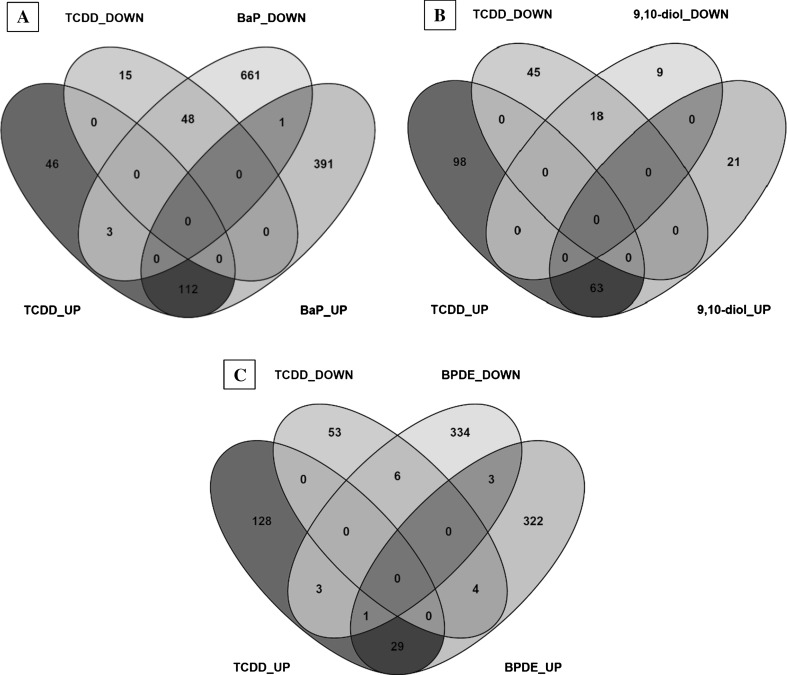
Venn diagrams representing the number of differentially expressed genes (DEGs) in common between TCDD and **a** BaP, **b** 9,10-diol and **c** BPDE considering direction of modulation (up- or down-regulated)

BaP and 9,10-diol treatments generated gene expression profiles which showed the highest similarity to those induced by TCDD (Supplementary Figure 2). Approximately 70 % of total gene modulation induced by 9,10-diol was shown to be overlapping with TCDD-induced gene expressions (Fig. 3); regulation of AhR, HIF-1α, AP-1 and Nrf2 transcriptional batteries was among the affected processes. Pathway analysis of genes overlapping between BaP and TCDD exposures also showed similar responses, covering all aforementioned pathways and an additional set of pathways (e.g., “RANKL-RANK pathway,” “ATF-2 transcription factor pathway,” “Regulation of androgen receptor activity,” among others) (Supplementary Data 2). Despite this overlap, a large gene set remained after subtraction of the TCDD expression signature, and even after removal of the genotoxic signature (i.e., BPDE-related gene expression), a group of almost 800 genes was found to be exclusively modulated by BaP treatment (Supplementary Data 3). These particular genes were found to play a role in several pathways, related to amino acid, lipid and cholesterol metabolism, as well as to p53, oxidative stress and apoptotic signaling.

### Promotion toward HCC status

In order to evaluate the contribution of BaP and/or its metabolite to the onset of hepatocarcinogenesis, we compared DEGs for each compound and time of exposure to a transcriptome signature from liver biopsies of ten HCC patients compared to healthy patients (Caiment et al. 2014). The signature comprises 8934 genes which are consistently regulated in all 10 patients (up- or down-regulated among all samples), regardless of any fold change or *p* value cutoff. A union list of DEGs from all time points for each compound shows that BaP (208 DEGs) and BPDE (130) induced the majority of nonredundant genes with the same direction of expression as HCC, followed by TCDD (36) and 9,10-diol (23).

BaP presented the largest unique set of induced gene expressions in overlap with the HCC signature, not shared by any other BaP metabolite or TCDD. Pathways associated with HCC and involved in lipid metabolism (e.g., metabolism of lipid and lipoproteins and arachidonic metabolism), as well as genes related to p53 response, were significantly affected after 12 and 18 h of BaP exposure. BPDE also caused a myriad of unique gene expression modifications overlapping with the HCC signature, which are linked to cell cycle (e.g., G1/S transition, cell cycle, M/G1 transition), metabolism (e.g., aminosugars metabolism, hexose/glucose transport) and DNA metabolism. Genes upregulated by 9,10-diol and over-expressed in HCC were also found among the BaP-induced DEGs (e.g., *CYP24A1*, *TXNRD1*) and also in the TCDD-associated gene set (*NQO1*, *GCNT3* and *STC2*).

 Interestingly, TCDD exclusively modulated a cluster of 13 genes (i.e., not found in any other treatment) into the same direction as in the HCC signature, including membrane trafficking regulators (*OPTN*), nuclear-cytoplasmic transporters (*MVP*) and genes encoding structural proteins (*COMP*, *TNNI2* and *MAPT*).

Also, DNA damage seems to influence repression/overexpression of HCC features. From the 114 genes significantly correlated to DNA adduct levels, 15 were also found in the HCC signature demonstrating the same direction of expression. Genes that were expressed in association with DNA damage but are not directly linked to repair or cell cycle arrest were found to encode for transcriptional regulators (*ZNF79*), enzymes from primary metabolism cluster (*TIGAR*), solute carriers (*MMD*, *SLC6A14*) and others; repressed genes included *MSRA* and *ZHX3*.

## Discussion

In this study, we evaluated gene expression changes in a liver cell model in response to BaP and three of its metabolites. Primary metabolites of BaP include epoxides, dihydrodiols, phenols and quinones, besides a substantial amount of transient intermediary metabolites (Miller and Ramos 2001). To assess the contribution of stable metabolites to the overall BaP-related toxicity, we analyzed alterations induced by a phenol (3-OH–BaP), a dihydrodiol (9,10-diol) and a diol epoxide (BPDE). BPDE being the major DNA adduct-forming metabolite, 3-OH–BaP and 9,10-diol are generated in a considerable amount and are described as substrates for further metabolic conversion, resulting in catechols and quinones (Miller and Ramos 2001). In order to grasp the potential contribution of these non-genotoxic metabolites to BaP toxicity, we separately evaluated transcriptomic effects of TCDD, a potent, non-genotoxic AhR agonist with carcinogenic properties, to compare patterns of gene expression modulation.

BaP-exposed cells showed DNA adduct formation at levels comparable to BPDE-treated cells (Fig. 1); while BaP requires multiple enzymatic steps, and consequently, more time to be converted into BPDE, direct exposure to this metabolite resulted in almost instant DNA adduction. In spite of several studies describing HepG2 as a model with limited biotransformation capacity (Jover et al. 1998; Wilkening et al. 2003), our results showed that HepG2 cells were indeed capable of activating these drug-metabolizing enzymes and fully responded to the BaP stimulus, resulting in extensive modulation of gene expression.

3-OH–BaP, considered the major hydroxylated metabolite resulting from BaP metabolism, had limited effect on gene expression of HepG2 cells, inducing only a few genes which appeared mainly involved in adaptive responses to oxidative stress: *AKR1B10*, *SRXN1* and *GCLM*. Although this response may seem compatible with the hypothetical formation of quinones, these compounds can be rapidly interconverted into more unstable compounds, and thus, these alterations may be a result of a transient stimulus. Also, even though a previous report showed 3-OH–BaP to be a potent ligand for the estrogen receptor (Charles et al. 2000; Fertuck et al. 2001), no alteration in mRNA levels of this particular gene was observed in this study, possibly as a result of low dose used (IC_20_).

As a non-genotoxic compound, mechanisms of TCDD-induced carcinogenesis are not yet fully understood, but increase in ROS formation, hormonal imbalance and alteration of cell proliferation and differentiation were reported as outcomes from TCDD exposure (Hernández et al. 2009), not to mention the still unclear role played by AhR in neoplastic transformation (Boffetta et al. 2011). Besides the broad effects in gene expression—which resulted in alterations of several TF networks, including ATF-2, AP-1, HIF-1α, p53—9,10-diol stood out due to the high similarity of its induced gene expression modifications to those caused by TCDD exposure and due to its ability to act as an AhR agonist. It was capable of inducing the AhR targets *CYP1A1* and *NQO1* at levels comparable to BaP and even TCDD (2 and 12 times; 2 and 13 times, respectively). Moreover, activation of Nrf2 transcription battery and upregulation of antioxidant enzymes indicate that 9,10-diol and/or its downstream metabolites induce oxidative damage. Thus, 9,10-diol and probably other dihydrodiols may play a key role in early BaP toxicity through non-genotoxic (and possibly AhR-mediated) routes, promoting oxidative stress and amplifying its harmful effects by positively regulating its conversion into toxic metabolites.

However, most transcriptomic alterations are likely to be related to genotoxic mechanisms, since BaP and BPDE modulated the largest number of DEGs. Interestingly, although both treatments resulted in DNA damage, only BPDE (at 6 h of exposure) induced a distinctive cluster containing genes from the DNA repair machinery. This is noteworthy since in mammalian cells induction of such genes is quite limited: First, activation and/or enhancement of repair activity relies mostly on posttranslational modifications (PTMs); second, transcriptional activation is only observed in a narrow genotoxicant dose range, since extensive DNA lesions can block their transcription (Christmann and Kaina 2013). In addition to DNA repair pathways, only BPDE activated the pentose phosphate pathway (PPP) and the ATM-dependent DNA damage response. Both pathways were shown to closely cooperate in response to double-strand breaks (DSB) in DNA: ATM recruits and activates the PPP pathway, which increases the input of nucleotides to be used during the replacement of damaged DNA bases (Cosentino et al. 2011). Thus, our results suggest that in contrast to BaP, where the cell can quickly adapt to increasing levels of genotoxicant, abrupt stimuli induced by BPDE have resulted in extensive DNA damage which triggered enhanced expression of genes necessary for DNA repair in order to protect the cell against future challenges (Lei et al. 2007).

Despite this singularity, transcriptional repression was observed both during BaP and BPDE challenges, in particular when high levels of DNA adduction were formed. BaP (12 h) and BPDE (6 h) shared a total of 157 downregulated genes, from which a small gene cluster related to “regulation of RhoA activity,” “Rho GTPase cycle” and GTPase molecular functions was found. Rho GTPases are mainly related to cell morphology and regulation of actin cytoskeleton, but there is also strong evidence of their roles in gene expression, cell proliferation and survival (Sahai and Marshall 2002). Repression of such genes may be indicative of cell cycle arrest and increased apoptotic response following extensive DNA damage, but may also be a consequence of transcriptional blockage due to the presence of repair machinery at these genomic sites.

Interestingly, genes associated with BaP-/BPDE-induced DNA lesions were also found to be similarly expressed in the HCC signature. Methionine sulfoxide reductase A (*MSRA*) and *ZHX3,* both down-regulated in the HCC signature and negatively correlated with adduct formation, were recently described as neoplastic markers in liver (Lei et al. 2007; Yamada et al. 2009). In contrast, expression of *TIGAR*, a p53-regulated multifunctional protein with a wide range of activities (including aid to DNA repair, organelle degradation, sugar metabolism shunt from glycolysis into pentose phosphate pathway, among others), was found to be up-regulated in HCC and positively correlated with DNA lesions induced by BPDE. *TIGAR* overexpression is also linked to increased glycolytic rate and decreased cell death, important (emergent) cancer hallmarks (Bensaad et al. 2006). Thus, it is evident that some genes engaged (in)directly in DNA repair may perform other intracellular roles, thereby influencing progression toward HCC.

Although BaP and BPDE specifically induced the largest cluster of overlapping genes with the HCC signature, fortuitous gene overlay is to be expected when comparing large expression datasets. Thus, we applied VennMapper to the HCC signature and our DEGs list: Gene expression deregulated by BaP (at 12 and 18 h of exposure), by 9,10-diol (at 12 h) and by BPDE (at18 h) surpassed the threshold of significance, mostly with regard to upregulated genes (Supplementary Figure 3). Interestingly, extensive gene modulation caused by TCDD did not show a significant similarity to HCC. This phenomenon also discards the suggestion of false positives or overlapping solely by chance. Although high levels of AhR mRNA have been described in many cancers and play a major role in the onset of rodent hepatocarcinomas (Safe et al. 2013), we did not find any relation between TCDD-mediated AhR activation and the in vivo HCC signature.

 Pathway analysis (Table 1) showed “hemostasis,” “NF-KB signaling” and “cytokine–cytokine receptor interaction” as processes from upregulated genes and “metabolism”/“metabolism of lipids and lipoproteins” from downregulated genes—with BaP as the main modulator. Notably, mid-term and late-term BaP treatments resulted in impairment of several lipid biosynthetic processes, both general and specific. Enzyme-coding genes from several reactions along the cholesterol biosynthetic pathway were found to be downregulated, including *HMGSC1* and *DHCR24*, which catalyze the first and the last enzymatic steps, and *HMGCR*, a rate-limiting enzyme from mevalonate pathway. *HMGCR* and other repressed genes such as *LSS* and *GPAM* (triacylgliceride metabolism) are targeted by the sterol regulatory element-binding protein and TF *SREBF*-*1*, also repressed. This is similar to previous findings using HepG2 as well as other liver models, i.e., cultured primary human hepatocytes and HepaRG subjected to BaP exposure, which showed “cholesterol biosynthesis” as a downregulated process (van Delft et al.; Jetten et al. 2013). BaP was recently pointed as the cause of decreased plasma concentrations of high-density lipoprotein cholesterol (HDLc) in smokers, suppressing apolipoprotein A-I mRNA levels in an AhR-dependent mechanism (Naem et al. 2012). In fact, 5 apolipoprotein genes appeared as differentially expressed compared to controls, 3 of them being repressed (*APOC3*, *APOA5* and *APOB*). Decreased plasma levels of not only (apo)lipoproteins, but also triglycerides, cholesterol and free fatty acids are often observed in HCC patients (Jiang et al. 2006). Thus, our results suggest that BaP exposure may have distressful effects on lipid metabolism, given the significant similarity to a condition where such alterations reflect hepatic impairment. Furthermore, these processes do not seem to be mediated by AhR or entirely by DNA damage. After subtraction of BPDE-related and TCDD-related (i.e., GTX and NGTX paths) gene signatures, these processes still appeared in the remaining BaP-associated gene set (Supplementary Data 3), which points to paths independent of AhR activation or DNA damage and is an indicative of a complex response of (groups of) metabolites or even BaP itself.

**Table 1:** Genes significantly altered by at least 1.5-fold in response to BaP with same direction of regulation in HCC signature set and their respective biological processes

Pathway	Up		Down	
Hemostasis	CD58	DGKG	GATA6	
	SLC3A	ITGA2		
	LRP8	MAFF		
	PLAU			
NF- κβ signaling	**ICAM1**	**RELB**	–	
	TAB 3	BIRC3		
	TNFRSF11A			
Cytokine–cytokine receptor interaction	CCL20	IL11	–	
	TNFRSF10B	TNFRSF12A		
	TNFRSF21			
Metabolism	SLC25A28	TALDO	CA5A	BCKDHB
	GLS	B3GNT3	BHMT	ALDH6A1
	SMOX	NQO1	ENO3	ADH6
Metabolism of lipids and lipoproteins	ABCC1	ACER3	LIPC	LCAT
	TXNRD1	GLA	PNPLA3	SREBF1
	NFYA	**CYP24A1**	CYP8B1	GPAM
			LSS	MSMO1
			PEMT	EPHX2
			AKR1D1	

Genes in bold were also modulated in exposure(s) to metabolites

Furthermore, BaP and metabolites were also shown to relate to HCC by activating cancer-related transcription factor networks involved in proliferation and inflammation (e.g., AP-1, HIF-1, ATF-2 and NF-κβ). Interestingly, a clear regulation loop for SNAI-CDH1 is observed only in BaP-exposed cells, with increased expression of SNAI after 6 and 12 h, following decreased expression of CDH1 at 12 and 18 h. SNA1 is induced by HIF-1 and acts as potent repressor of CDH1 (whose protein product is a mediator of cell–cell adhesion) and inducer of metalloproteinases involved in tissue modeling (Semenza 2003; Zheng et al. 2013), which may also point a role on cell migration and metastasis.

In conclusion, here we show that BaP is capable of promoting a HCC-like scenario in HepG2 through AhR-mediated and genotoxic mechanisms, with impairment of important liver attributions and induction of notoriously poor prognostic features in neoplasms. Although these may seem transient alterations, it is important to consider that BaP omnipresence in environment and food imposes constant accumulation of exposure. In a long-term context, harmful activities of BaP and its metabolites may reach a point where alterations may be irreversible possibly initiating HCC. Thus, the BaP-induced transcriptomic changes observed in this study and framed into a neoplastic scenario provide important information on the mechanisms of BaP-induced hepatocarcinogenesis.

## Electronic supplementary material

Below is the link to the electronic supplementary material.

Supplementary Figure 1 Similarity scores (Z score) for gene expression sets from BaP and TCDD and BaP and its metabolites. Clusters above gray line (equal to 1.96) are statistically significant and indicate that gene overlap is not due to fortuity. (PDF 7 kb)

Supplementary Figure 2 Similarity scores (Z score) for gene expression sets from BaP(-metabolites) and TCDD. Clusters above gray line (equal to 1.96) are statistically significant and indicate that gene overlap is not due to fortuity. (PDF 7 kb)

Supplementary Figure 3 Similarity scores (Z score) for genes significantly modulated by BaP(-metabolites) and TCDD treatments present in the HCC signature set with same direction of regulation. Clusters above gray line (equal to 1.96) are statistically significant and indicate that gene overlap is not due to fortuity. (PDF 128 kb)

Supplementary material 4 (XLSX 339 kb)

Supplementary material 5 (PDF 227 kb)

Supplementary material 6 (PDF 244 kb)

## References

[CR1] Altekruse SF, McGlynn KA, Reichman ME (2009). Hepatocellular carcinoma incidence, mortality, and survival trends in the United States from 1975 to 2005. J Clin Oncol.

[CR2] Bensaad K, Tsuruta A, Selak MA (2006). TIGAR, a p53-inducible regulator of glycolysis and apoptosis. Cell.

[CR3] Boffetta P, Mundt KA, Adami H-O (2011). TCDD and cancer: a critical review of epidemiologic studies. Crit Rev Toxicol.

[CR4] Caiment F, Tsamou M, Jennen D, Kleinjans J (2014). Assessing compound carcinogenicity in vitro using connectivity mapping. Carcinogenesis.

[CR5] Charles GD, Bartels MJ, Zacharewski TR (2000). Activity of benzo[a]pyrene and its hydroxylated metabolites in an estrogen receptor-alpha reporter gene assay. Toxicol Sci.

[CR6] Christmann M, Kaina B (2013). Transcriptional regulation of human DNA repair genes following genotoxic stress: trigger mechanisms, inducible responses and genotoxic adaptation. Nucleic Acids Res.

[CR7] Cosentino C, Grieco D, Costanzo V (2011). ATM activates the pentose phosphate pathway promoting anti-oxidant defence and DNA repair. EMBO J.

[CR8] El-Serag HB, Marrero JA, Rudolph L, Reddy KR (2008). Diagnosis and Treatment of Hepatocellular Carcinoma. Gastroenterology.

[CR9] Farazi PA, DePinho RA (2006). Hepatocellular carcinoma pathogenesis: from genes to environment. Nat Rev Cancer.

[CR10] Fertuck KC, Matthews JB, Zacharewski TR (2001). Hydroxylated benzo[a]pyrene metabolites are responsible for in vitro estrogen receptor-mediated gene expression induced by benzo[a]pyrene, but do not elicit uterotrophic effects in vivo. Toxicol Sci.

[CR11] Godschalk RWL, Maas LM, Van Zandwijk N (1998). Differences in aromatic-DNA adduct levels between alveolar macrophages and subpopulations of white blood cells from smokers. Carcinogenesis.

[CR12] Hernández LG, van Steeg H, Luijten M, van Benthem J (2009). Mechanisms of non-genotoxic carcinogens and importance of a weight of evidence approach. Mutat Res.

[CR13] Hockley SL, Arlt VM, Brewer D (2006). Time- and concentration-dependent changes in gene expression induced by benzo(a)pyrene in two human cell lines, MCF-7 and HepG2. BMC Genom.

[CR14] IARC (1983). Polynuclear Aromatic compounds, Part 1, Chemical.

[CR15] IARC (2012). Benzo[a]pyrene. IARC monogr. eval. carcinog. risks to humans.

[CR16] Jetten MJA, Kleinjans JCS, Claessen SM (2013). Baseline and genotoxic compound induced gene expression profiles in HepG2 and HepaRG compared to primary human hepatocytes. Toxicol Vitr.

[CR17] Jiang J, Nilsson-Ehle P, Xu N (2006). Influence of liver cancer on lipid and lipoprotein metabolism. Lipids Health Dis.

[CR18] Jover R, Bort R, Gómez-Lechón MJ, Castell JV (1998). Re-expression of C/EBP alpha induces CYP2B6, CYP2C9 and CYP2D6 genes in HepG2 cells. FEBS Lett.

[CR19] Kamburov A, Stelzl U, Lehrach H, Herwig R (2013). The ConsensusPathDB interaction database: 2013 update. Nucleic Acids Res.

[CR20] Knasmüller S, Parzefall W, Sanyal R (1998). Use of metabolically competent human hepatoma cells for the detection of mutagens and antimutagens. Mutat Res Mol Mech Mutagen.

[CR21] Kuper H, Tzonou A, Kaklamani E (2000). Tobacco smoking, alcohol consumption and their interaction in the causation of hepatocellular carcinoma. Int J Cancer.

[CR22] Lee YCA, Cohet C, Yang YC (2009). Meta-analysis of epidemiologic studies on cigarette smoking and liver cancer. Int J Epidemiol.

[CR23] Lei K-F, Wang Y-F, Zhu X-Q (2007). Identification of MSRA gene on chromosome 8p as a candidate metastasis suppressor for human hepatitis B virus-positive hepatocellular carcinoma. BMC Cancer.

[CR24] Magkoufopoulou C, Claessen SMH, Jennen DGJ (2011). Comparison of phenotypic and transcriptomic effects of false-positive genotoxins, true genotoxins and non-genotoxins using HepG2 cells. Mutagenesis.

[CR25] Miller KP, Ramos KS (2001). Impact of cellular metabolism on the biological effects of benzo[a]pyrene and related hydrocarbons. Drug Metab Rev.

[CR26] Naem E, Alcalde R, Gladysz M (2012). Inhibition of apolipoprotein A-I gene by the aryl hydrocarbon receptor: a potential mechanism for smoking-associated hypoalphalipoproteinemia. Life Sci.

[CR27] Safe S, Lee S-O, Jin U-H (2013). Role of the aryl hydrocarbon receptor in carcinogenesis and potential as a drug target. Toxicol Sci.

[CR28] Sahai E, Marshall CJ (2002). RHO-GTPases and cancer. Nat Rev Cancer.

[CR29] Semenza GL (2003). Targeting HIF-1 for cancer therapy. Nat Rev Cancer.

[CR30] Smid M, Dorssers LCJ, Jenster G (2003). Venn mapping: clustering of heterologous microarray data based on the number of co-occurring differentially expressed genes. Bioinformatics.

[CR31] Van Delft JHM, van Agen E, van Breda SGJ (2004). Discrimination of genotoxic from non-genotoxic carcinogens by gene expression profiling. Carcinogenesis.

[CR32] Van Delft JHM, Mathijs K, Staal YCM (2010). Time series analysis of benzo[a]pyrene-induced transcriptome changes suggests that a network of transcription factors regulates the effects on functional gene sets. Toxicol Sci.

[CR34] Van Delft J, Gaj S, Lienhard M (2012). RNA-seq provides new insights in the transcriptomic responses induced by the carcinogen benzo(a)pyrene. Toxicol Sci.

[CR33] Wilkening S, Stahl F, Bader A (2003). Comparison of primary hepatocytes and hepatome cell line HepG2 with regard to their biotransformation properties. Drug Metab Dispos.

[CR35] Yamada K, Ogata-Kawata H, Matsuura K (2009). ZHX2 and ZHX3 repress cancer markers in normal hepatocytes. Front Biosci.

[CR36] Zheng SS, Chen XH, Yin X, Zhang BH (2013). Prognostic significance of HIF-1? Expression in hepatocellular carcinoma: A meta-analysis. PLoS One.

