# Supplementary figures and images for: New insights into BaP-induced toxicity: role of major metabolites in transcriptomics and contribution to hepatocarcinogenesis
Source: Arch Toxicol. 2015 Aug 4;90:1449–58. doi: 10.1007/s00204-015-1572-z (PMC4873527; doi:10.1007/s00204-015-1572-z)

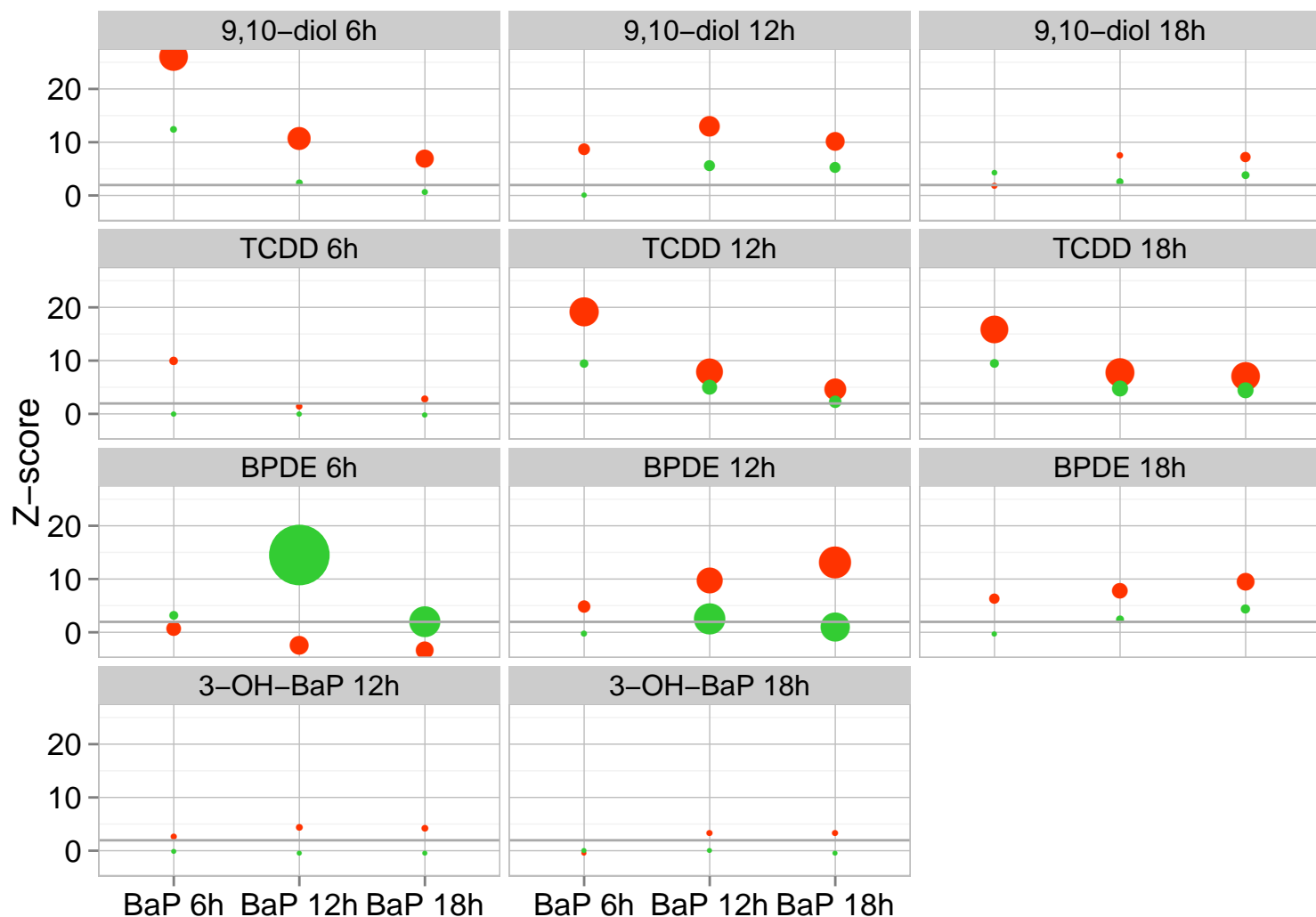

Supplement: Supplementary file 1 — Similarity scores (Z score) for gene expression sets from BaP and TCDD and BaP and its metabolites. Clusters above gray line (equal to 1.96) are statistically significant and indicate that gene overlap is not due to fortuity. (PDF 7 kb) [file 204_2015_1572_MOESM1_ESM.pdf]

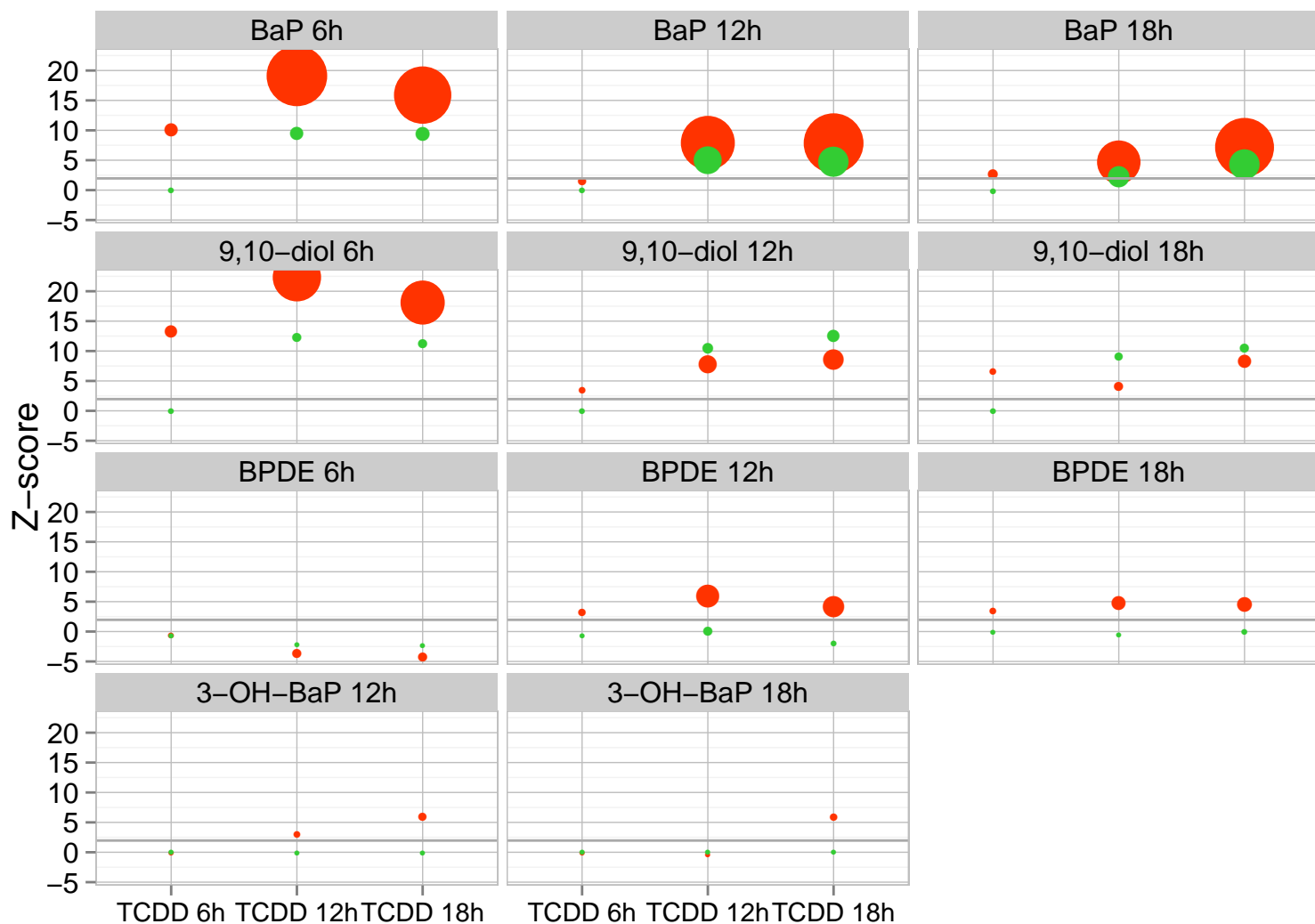

Supplement: Supplementary file 2 — Similarity scores (Z score) for gene expression sets from BaP(-metabolites) and TCDD. Clusters above gray line (equal to 1.96) are statistically significant and indicate that gene overlap is not due to fortuity. (PDF 7 kb) [file 204_2015_1572_MOESM2_ESM.pdf]

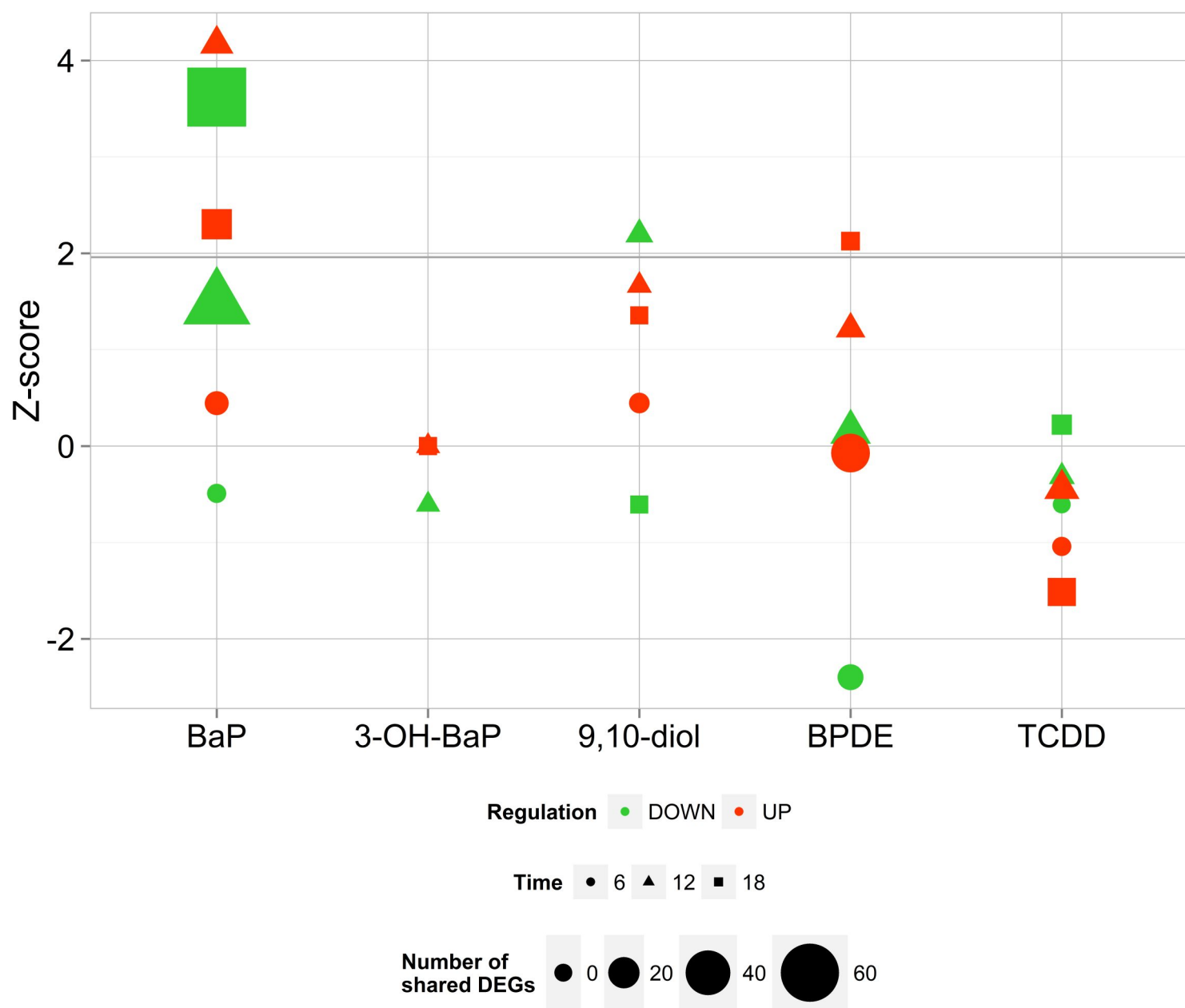

Supplement: Supplementary file 3 — Similarity scores (Z score) for genes significantly modulated by BaP(-metabolites) and TCDD treatments present in the HCC signature set with same direction of regulation. Clusters above gray line (equal to 1.96) are statistically significant and indicate that gene overlap is not due to fortuity. (PDF 128 kb) [file 204_2015_1572_MOESM3_ESM.pdf]

### 1. Pathways from overlapped genes between BaP(-metabolites) and TCDD

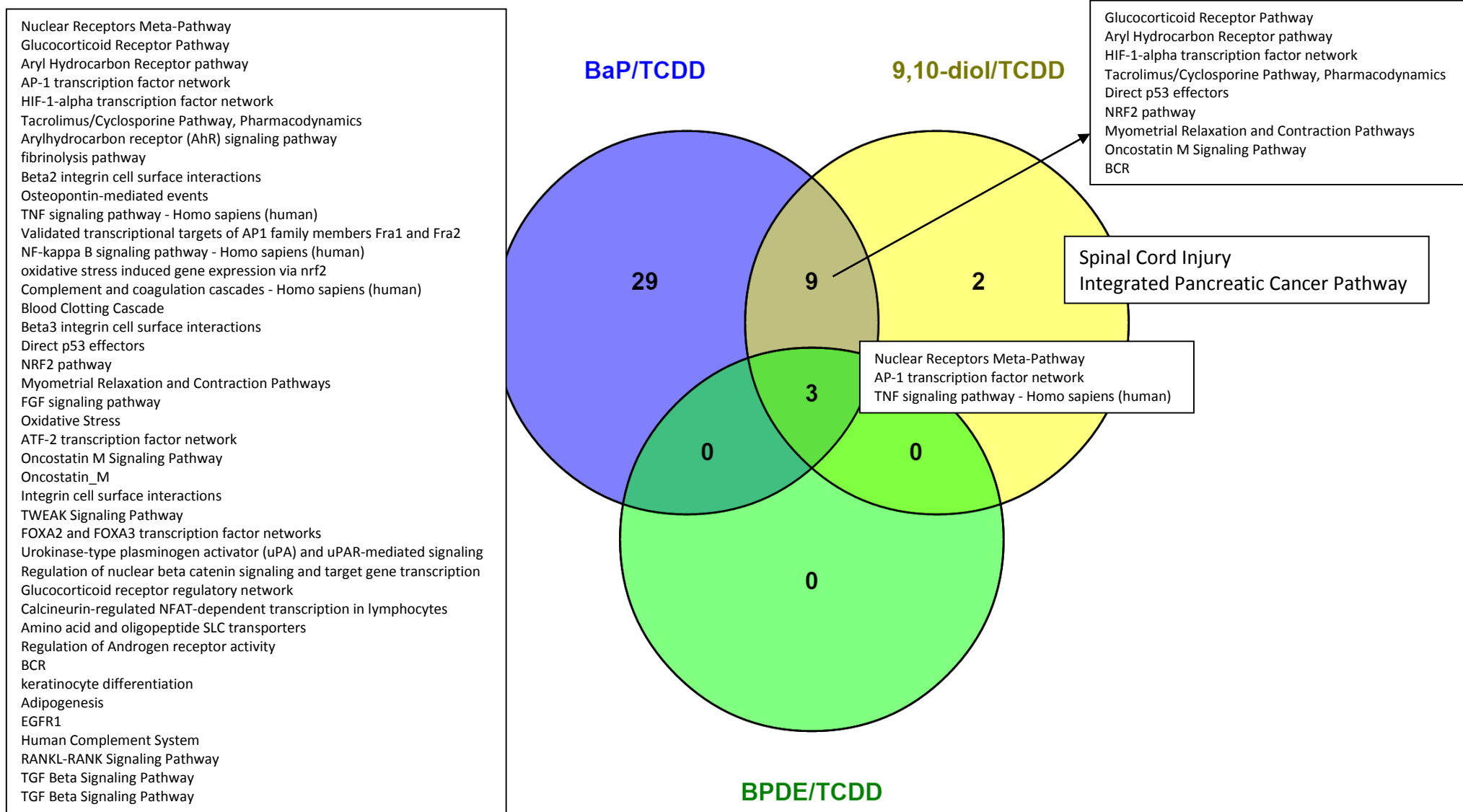

Supplement: Supplementary file 5 — Supplementary material 5 (PDF 227 kb) [file 204_2015_1572_MOESM5_ESM.pdf]
