# Supplementary material for: New insights into BaP-induced toxicity: role of major metabolites in transcriptomics and contribution to hepatocarcinogenesis
Source: Arch Toxicol. 2015 Aug 4;90:1449–58. doi: 10.1007/s00204-015-1572-z (PMC4873527; doi:10.1007/s00204-015-1572-z)
Supplement: Supplementary file 6 — Supplementary material 6 (PDF 244 kb) [file 204_2015_1572_MOESM6_ESM.pdf]

1. **Overlapping genes between BaP and BPDE after subtraction of TCDD gene signature (DEGs with same direction of expression)**

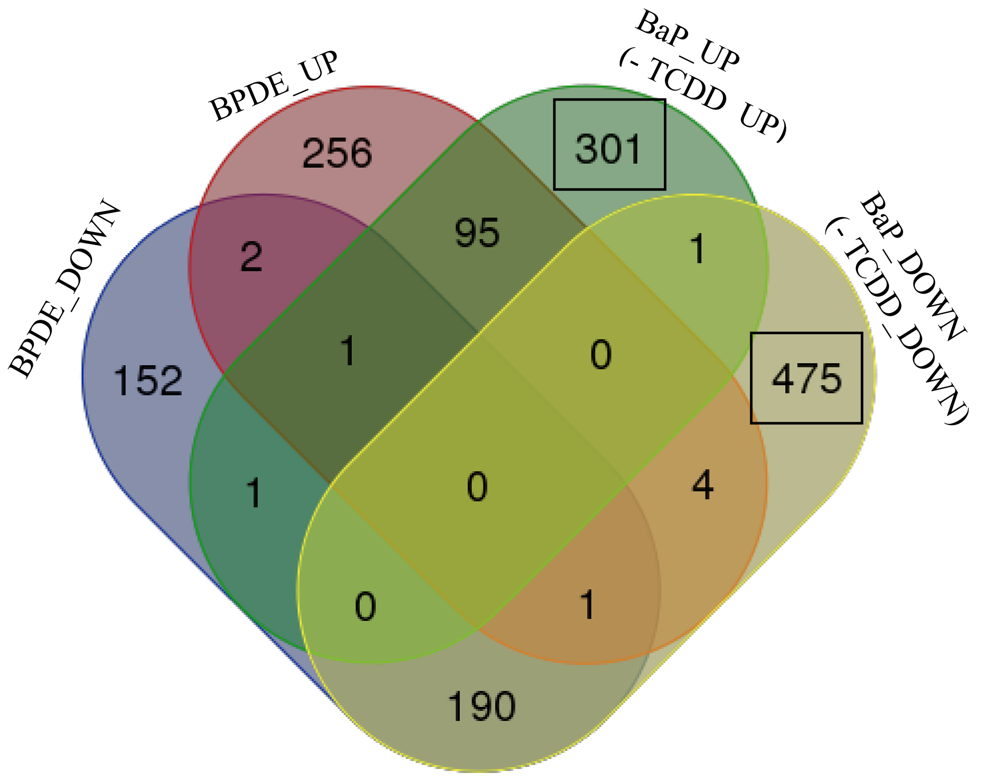

1. **Pathway analysis from genes exclusively regulated in BaP treatment**

Pathway	q-value	Database
**Up-regulated**		
Direct p53 effectors	0.000344	PID
NRF2 pathway	0.0013	Wikipathways
Oxidative stress	0.00459	Wikipathways
Nuclear receptors meta-pathway	0.00507	Wikipathways
Apoptosis modulation and signaling	0.0065	Wikipathways
P53 signaling pathway	0.00797	KEGG
Validated transcriptional targets of deltaNp63 isoforms	0.00797	PID
Osteoclast differentiation	0.00823	KEGG
**Down-regulated**		
Metabolism	4.03E-07	Reactome
**Cholesterol biosynthesis**	**5.89E-07**	**Reactome**
**Cholesterol Biosynthesis**	**1.54E-05**	**Wikipathways**
**SREBF and miR33 in cholesterol and lipid homeostasis**	**5.15E-05**	**Wikipathways**
Glycine, serine and threonine metabolism - Homo sapiens (human)	6.88E-05	KEGG
**Steroid biosynthesis - Homo sapiens (human)**	**6.88E-05**	**KEGG**
Metabolism of amino acids and derivatives	0.000249	Reactome
Alanine, aspartate and glutamate metabolism - Homo sapiens (human)	0.000265	KEGG
**Metabolism of lipids and lipoproteins**	**0.000334**	**Reactome**
Nitrogen metabolism - Homo sapiens (human)	0.000402	KEGG
Amino acid synthesis and interconversion (transamination)	0.000402	Reactome
Terpenoid backbone biosynthesis - Homo sapiens (human)	0.00116	KEGG
**Activation of gene expression by SREBF (SREBP)**	**0.00215**	**Reactome**
Glyoxylate and dicarboxylate metabolism - Homo sapiens (human)	0.00258	KEGG
Butanoate metabolism - Homo sapiens (human)	0.00307	KEGG
Regulation of cholesterol biosynthesis by SREBP (SREBF)	0.0051	Reactome
**SREBP signalling**	**0.00546**	**Wikipathways**

Trans-sulfuration and one carbon metabolism	0.00663	Wikipathways
Aurora A signaling	0.00663	PID
Valine, leucine and isoleucine degradation - Homo sapiens (human)	0.00668	KEGG
Endochondral Ossification	0.00683	Wikipathways
Activation of Gene Expression by SREBP (SREBF)	0.00716	Wikipathways
Signaling by Wnt	0.00736	Reactome
